# How Robust Are Our Care Plans for Patients Detained Under Community Compulsory Treatment Orders?

**DOI:** 10.1192/bjo.2025.10534

**Published:** 2025-06-20

**Authors:** Louisa Adam, Susannah Houston, Suzanne Galloway, Andrew Donaldson, Martin Carlin

**Affiliations:** NHS Lanarkshire, Glasgow, United Kingdom

## Abstract

**Aims:** The Mental Welfare Commission (MWC) released a report in February 2024 recommending the use of audit to ensure good clinical practice in the use of community Compulsory Treatment Orders (cCTO) as part of the Mental Health (Care and Treatment) (Scotland) Act 2003 (MHA). One particular area of concern was the use of care plans under section 76 of the Act.

An audit was performed across NHS Lanarkshire Mental Health services to determine if all patients on cCTOs had Section 76 care plans in place that were valid and compliant with the minimum standards set out by the MWC.

**Methods:** Medical records administration staff were contacted across all of the psychiatry specialities within the health board, to supply a list of patients on cCTOs. Their electronic medical records were reviewed and relevant data collated by the authors to determine if the appropriate paperwork was in place, was valid, and met the minimum standards, as set out by the MWC.

**Results:** Within NHS Lanarkshire, there were 89 patients on cCTOs. 87 of these had a Section 76 care plan in place, though one of these was considered invalid.

Only 24% of the care plans were found to meet all of the minimum standards. There was noted to be a high degree of variability in which of the minimum standards were met, how the care plans were documented and the quality of the information contained within them, across the specialties and between individual psychiatrists.

**Conclusion:** This was the first audit looking at cCTO Section 76 care plans carried out in NHS Lanarkshire. It demonstrated there is a need for standardisation of these care plans across mental health services, to ensure that as a minimum, all statutory information is documented.

Recommendations from the audit included the use of a proforma to capture the information required to meet the minimum standards, as well as provide prompts for additional information to improve the quality of the care plans. It has also been recommended that each psychiatry specialty sets up their own annual audit of care plans, and an audit tool for this has been provided.

